# Pulmonary Herpes Simplex Virus Reactivation in Invasively Mechanically Ventilated Patients With COVID‐19 in Intensive Care Unit

**DOI:** 10.1002/jmv.71170

**Published:** 2026-10-07

**Authors:** Bastien Zarlenga, Florent Wallet, Alexandre Gaymard, Anne‐Claire Lukaszewicz, Gregory Destras, Christophe Ginevra, Bruno Simon, Jennifer Giorgi, Muriel Rabilloud, Salomé Avenas, Alexis Trecourt, Geneviève Billaud, Florence Morfin, Jean‐Christophe Richard, Emilie Frobert

**Affiliations:** ^1^ Hospices Civils de Lyon, Laboratoire de Virologie, Institut des Agents Infectieux, Groupement Hospitalier Nord Lyon France; ^2^ Hospices Civils de Lyon, Médecine Intensive et Réanimation, Centre Hospitalier Universitaire Lyon Sud Université Claude Bernard Lyon 1 Lyon France; ^3^ Virpath, Centre International de Recherche en Infectiologie, INSERM U1111, CNRS UMR5308, ENS Lyon Université Claude Bernard Lyon 1 Lyon France; ^4^ Anesthesia and Critical Care Medicine Department, Hopital Edouard Herriot, Hospices Civils de Lyon Université de Lyon, EA 7426 « Pathophysiology of Injury‐Induced Immunosuppression », (Université Claude Bernard Lyon 1—Hospices Civils de Lyon—bioMérieux) Lyon France; ^5^ GenEPII Sequencing Platform, Institut des Agents Infectieux, Hospices Civils de Lyon Lyon France; ^6^ Laboratoire de Bactériologie, Centre National de Référence des Légionelles, Institut des Agents Infectieux, Hospices Civils de Lyon, Groupement Hospitalier Nord Lyon France; ^7^ Hospices Civils de Lyon, Pôle Santé Publique, Service de Biostatistique et Bioinformatique Lyon France; ^8^ Université Lyon 1 Villeurbanne France; ^9^ CNRS, UMR 5558, Laboratoire de Biométrie et Biologie Évolutive, Équipe Biostatistique‐Santé Villeurbanne France; ^10^ Université Claude Bernard Lyon 1 Service de Pathologie Multi‐Site, Hospices Civils de Lyon, Centre Hospitalier Lyon Sud Lyon France; ^11^ Université de Lyon, Université Claude Bernard Lyon 1, INSA‐Lyon, UJM‐Saint Etienne, CNRS, Médecine Intensive Réanimation, Hospices Civils de Lyon, Groupement Hospitalier Nord Lyon France

**Keywords:** antiviral resistance, COVID‐19, herpes simplex virus reactivation, intensive care unit, UL23 thymidine kinase, UL30 DNA polymerase

## Abstract

Patients in intensive care units (ICU) under mechanical ventilation, particularly those with SARS‐CoV‐2 infection, are highly susceptible to Herpes simplex virus (HSV) reactivation. Given the lack of data on the SARS‐CoV‐2 Omicron and Delta variants, this study aimed to assess the prevalence of pulmonary HSV reactivation in mechanically ventilated patients with SARS‐CoV‐2 pneumonia. The impact of HSV replication on SARS‐CoV‐2 replication, and conversely, was investigated, along with the susceptibility of HSV strains to antiviral therapies. This retrospective study included 381 mechanically ventilated ICU patients in Lyon. Overall, 103 patients (27.0%) experienced HSV reactivation, with a trend toward a higher prevalence among SARS‐CoV‐2‐positive patients (32.8%) compared to SARS‐CoV‐2‐negative (24.1%; *p* value = 0.058). HSV reactivation was significantly higher with the Delta variant (46.2%) compared to Omicron (27.1%; *p* value = 0.045); the Delta variant was associated with a 2.38‐fold increased risk of HSV reactivation. HSV‐positive patients exhibited a 1‐log lower SARS‐CoV‐2 viral load compared to HSV‐negative patients, a finding indicative of the natural course of SARS‐CoV‐2 infection followed by HSV reactivation, rather than viral interference. Unexpectedly, acyclovir resistance mutations appeared in the *UL23* gene in at least 13.6% of HSV‐1 strains in patients with an HSV load above 10^5^ copies/mL. Complementary phenotypic and genotypic approaches allowed to identify three novel substitutions in *UL30*: Q217R, D741A, and E756K, classified as polymorphisms. Further optimization is needed in the management of mechanically ventilated ICU patients with pulmonary HSV reactivation, especially regarding antiviral resistance testing.

## 1 Introduction

SARS‐CoV‐2 infection can lead to pneumonia associated with acute respiratory distress syndrome, requiring orotracheal intubation and invasive mechanical ventilation in the intensive care unit (ICU) [1]. Herpetic reactivation in the lower respiratory tract is common and has been documented in both immunocompromised and immunocompetent patients in the ICU [2, 3]. The Herpes simplex virus (HSV) can be detected in approximately 64% of mechanically ventilated patients, and its reactivation has been associated with prolonged ventilation duration [2, 4]. The role of HSV as either a causative agent of disease progression or a marker of an underlying disease remains debated. Reactivation of this pathogen can be explained, in part, by the immunosuppression; this immunosuppression may be related to pre‐existing underlying conditions or acquired in the ICU following acute events such as sepsis and the administration of immunosuppressive treatments [5]. Early during the COVID‐19 pandemic, HSV was the predominant respiratory viral co‐pathogen in invasively ventilated patients with SARS‐CoV‐2 pneumonia, along with the frequent co‐occurrence of HSV and Aspergillus infection [6]. In addition, HSV reactivations have been more frequently observed in patients with SARS‐CoV‐2‐related acute respiratory distress syndrome compared to critically ill patients [7]. Therefore, in the ICU, immunocompromised patients due to SARS‐CoV‐2 infection could provide a favorable environment for herpetic reactivation. This heightened vulnerability to herpetic reactivation, combined with patient immunosuppression and the current lack of consensus regarding the treatment of pulmonary herpetic disease, creates a favorable setting for the emergence of antiviral resistance.

The main available studies described pulmonary herpetic reactivation during the circulation of the SARS‐CoV‐2 Alpha and Beta variants, whereas only a few case reports are available for the Omicron variant [8]. In this context, the present study aimed to assess the prevalence of pulmonary HSV reactivation in mechanically ventilated patients with SARS‐CoV‐2 pneumonia due to Omicron and Delta variants during 2022. This study also investigated the potential impact of SARS‐CoV‐2 infection on HSV replication, as well as the susceptibility of HSV strains to antiherpetic treatments in the context of ICU‐related immunosuppression.

## 2 Materials and Methods

### 2.1 Patients and Clinical Samples

This single‐center retrospective study was conducted in the three ICUs of the *Hospices Civils de Lyon*, France, from January 1, 2022 to December 31, 2022, in invasively mechanically ventilated adult patients (≥ 18 years) who underwent SARS‐CoV‐2 and HSV analysis in low respiratory tract samples (LRTs). LRTs were defined as respiratory specimens corresponding to tracheal aspirates and bronchoalveolar lavages (BALs). LRTs were collected as part of the routine clinical care, and at the discretion of the clinician. Pregnant women and patients without PCR performed for both HSV and SARS‐CoV‐2 were excluded from the study. HSV reactivation in LRTs was defined as a single positive PCR for HSV, regardless of viral load.

### 2.2 Data Collection

Demographic, clinical, microbiological, and cytopathological data were retrospectively collected from the laboratory information management system (Clinisys Glims), local patient record database (Easily), and intensive care information system (IntelliSpace Critical Care and Anesthesia).

The following data were recorded: age, sex, Simplified Acute Physiology Score II (SAPS II) at admission, National Early Warning Score II (NEWS II) adjusted on chronic obstructive pulmonary disease (COPD), presence of an oxygenation support, ICU length of stay and mortality, use of corticosteroids, administration of antiviral treatments (acyclovir [ACV], foscarnet [FOS], ganciclovir/valganciclovir, combination of nirmatrelvir/ritonavir, remdesivir, and convalescent plasma), episodes of blood stream infection (BSI), HSV serological results, and presence of an immunosuppression. Patients were considered immunocompromised prior to ICU admission when they presented at least one the following conditions: being a solid organ transplant (SOT) and/or hematopoietic stem cell transplantation (HSCT) recipient; hematological malignancy; cancer with chemotherapy lasting < 6 months; use of immunotherapies such as rituximab < 6 months, anakinra, tocilizumab, ruxolitinib; uncontrolled HIV (stage 3); pancytopenia, and neutropenia.

### 2.3 HSV and SARS‐CoV‐2 Virological Analysis

#### 2.3.1 HSV and SARS‐CoV‐2 Viral Loads Analysis and Normalization in LRTs

HSV quantification was performed on ELITE BeGenius (ELITechGroup, Puteaux, France) using the HSV1/2 ELITe MGB assay (ELITechGroup). SARS‐CoV‐2 and cell quantification were achieved by RT‐qPCR using the COVID‐19 R‐GENE kit (BioMerieux, Marcy‐l'Etoile, France) on QuantStudio 5 Real‐Time PCR instrument (96 wells, 0.2 mL Block, Thermo Fisher Scientific, Waltham, MA, USA). All analyses were performed following the manufacturer's instructions. SARS‐CoV‐2 and HSV viral loads were normalized to the number of cells recovered to overcome virus‐load variations due to variability in sample collection (volume and cell number). The amount of respiratory epithelial cells was assessed through RNA quantification of the *hypoxanthine phosphoribosyltransferase 1* (*HPRT1*) housekeeping gene and reported as RNA copies/mL. Normalized HSV and SARS‐CoV‐2 viral loads were expressed as the number of viral copies/10^6^ cells according to the following formula: $\text{normalized viral load}\;=\frac{\text{number of HSV copies}/\text{mL}}{\text{number of copies of}\;\mathrm{HPRT}1\;}\;\cdot \;10^{6}$.

#### 2.3.2 HSV Resistance Testing

##### 2.3.2.1 Genotypic Analysis

HSV‐positive LRTs were sequenced in patients who had two specimens collected at least 7 days apart with an HSV viral load > 5 log copies/mL [4]. Next‐generation sequencing was carried out on *UL23* and *UL30* encoding Thymidine kinase (TK) and DNA polymerase (DNApol), as previously described [9]. All Illumina sequences have been deposited in the European Nucleotide Archive database (accession number PRJEB79652).

##### 2.3.2.2 Phenotypic Analysis

The chessboard method, using neutral red dye‐uptake assay, was performed following a positive viral culture in order to characterize non‐previously described substitutions regarding ACV and FOS polymorphism or resistance [10]. The inhibitory concentration 50 (IC50) cut‐off values were 6.5 μM for ACV and 350 μM for FOS [11]. IC50 values < 0.8 µM were reported as 0.8 µM. Substitutions that do not affect drug susceptibility were defined as natural polymorphisms, while those leading to drug resistance were defined as resistance substitutions. Mutations were categorized as of unknown significance (USM) when the phenotypic analysis could not be carried out. For viral strains showing USM with IC50 close to the cut‐off value, the phenotypic test was performed twice.

### 2.4 Cytopathological Analysis

A cytological analysis was performed to support the diagnosis of herpetic bronchopneumonia by demonstrating specific HSV nuclear inclusions, such as large, multinucleated cells with enlarged nuclei containing ground glass chromatin marginated and condensed at the periphery of the nuclei [2]. BALs were analyzed after monolayer preparation and Papanicolaou staining upon request from clinicians. HSV bronchopneumonia was defined as the combination of the two following criteria: presence of herpetic nuclear inclusions on cytology and a positive HSV PCR in LRTs.

### 2.5 Investigation of Other Co‐Infections

The investigation for bacterial, fungal, and viral co‐infections in LRTs was conducted as part of the standard patient care pathway upon the clinician's request. The detection of Influenza A and B, and respiratory syncytial virus (RSV) was carried out using Panther Fusion Flu A/B/RSV, while that of Adenovirus, Metapneumovirus as well as Rhinovirus was performed using Panther Fusion AdV/hMPV/RV Assay (Hologic, Marlborough, MA, USA). Bloodstream infection (BSI) was characterized as a bacterial or fungal infection detected on blood cultures.

### 2.6 Phylogenetic Analysis of SARS‐CoV‐2 Genome Among Mechanically Ventilated Patients

SARS‐CoV‐2 nucleotide sequences were obtained from the GISAID Epicov database [12]. Alignment was subsequently performed using Nexclade online tool relative to the original SARS‐CoV‐2 sequence (GenBank accession number MN908947.3) [13]. The maximum likelihood phylogenetic tree was inferred from the SARS‐CoV‐2 genome alignment after model selection (best fitting substitution model TIM + F + I according to BIC score [14]) using the IQ‐TREE web server [15]. Statistical robustness and reliability of the branching order within the tree were assessed by ultrafast bootstrapping (10,000 replicates) with IQ‐TREE [16]. The tree was then edited using FigTree version 1.4.4 software (http://tree.bio.ed.ac.uk/software/figtree/).

### 2.7 Statistical Analysis

Quantitative variables were expressed as median and interquartile range (IQR), and qualitative variables as count and percentage. The description was carried out in the overall population and according to the presence of HSV reactivation. Comparisons were carried out using *χ* ^2^ test or Fisher exact test for qualitative variables, and Student *t*‐test or Mann–Whitney test for quantitative variables. Univariate and multivariate logistic regression models were used to assess the association between HSV reactivation and the following factors: age, sex, presence of SARS‐CoV‐2 in LRTs and its variant when the data were available, underlying immunosuppression, use of corticosteroids, and use of extracorporeal membrane oxygenation (ECMO). The effect of each explanatory variable was quantified through the adjusted odds ratio (OR) with the associated 95% confidence interval (95% CI). Linear regression models were performed to assess the impact of the above‐mentioned factors on HSV and SARS‐CoV‐2 replication. To compare the normalized HSV viral load between patients with positive and negative SARS‐CoV‐2 PCR results, and conversely, to compare the normalized SARS‐CoV‐2 viral load according to HSV status, a Student's *t*‐test was performed. Spearman's rank coefficient was used to assess the correlation between non‐normalized (expressed in copies/mL) and normalized HSV viral loads (expressed in copies per million cells). A test was considered statistically significant for a *p* value < 0.05. All statistical analyses were performed using statistical software R, version 4.4.1.

### 2.8 Ethical Statement

In accordance with the current French law, non‐opposition was obtained from all included patients. Data storage and data analysis complied with legal and ethical standards, thereby safeguarding the privacy and rights of individuals whose data was being used (MR004 methodology as appropriate for French study; registration number 23‐5167). This study has been approved by the institutional review board of the *Hospices Civils de Lyon* on July 6, 2023.

## 3 Results

### 3.1 Study Population

During the study period, 1099 LRTs collected in ICUs from 454 different patients were sent to the virology laboratory for SARS‐CoV‐2 or HSV analysis. Among them, 381 patients (93.2%) met the inclusion criteria and were therefore included. Overall, 128 patients (33.6%) were SARS‐CoV‐2–positive, of whom 42 (32.8%) were HSV‐positive. Among the 253 SARS‐CoV‐2–negative patients, 61 (24.1%) were HSV‐positive (Figure 1).

**Figure 1:**
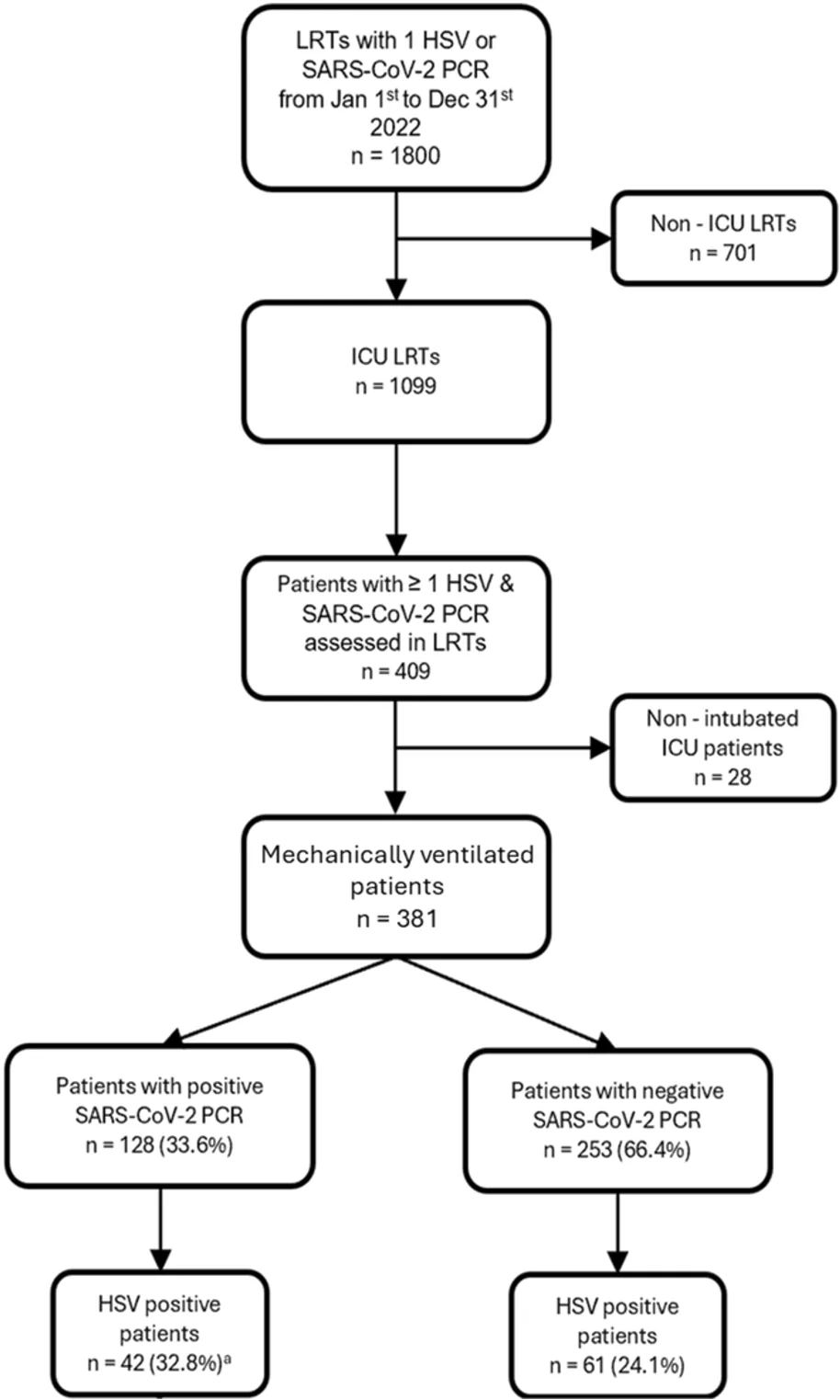
Flow chart of patients and LRTs from the study. ICU, intensive care unit; HSV, herpes simplex virus; LRTs: low respiratory tract samples; PCR, polymerase chain reaction; SARS‐CoV‐2, severe acute respiratory syndrome coronavirus 2.

#### 3.1.1 Characteristics at ICU Admission

The median age of the overall study population was 63.0 years (IQR 52.0–70.5), patients were mostly males (71.1%), and their median SAPS II at ICU admission was 49.5 (IQR 37.0–66.0). Overall, 157 patients (41.2%) were immunocompromised; while proportions of immunocompromised patients between the HSV‐positive and HSV‐negative patients were similar, the proportion of patients with a hematological malignancy was twice greater in the HSV‐negative group. All HSV‐positive patients had a positive anti‐HSV IgG serology prior to their ICU admission (Table 1).

**Table 1:** Characteristics of the study population at ICU admission.

	Overall population (*n* = 381)	HSV positive (*n* = 103)	HSV negative (*n* = 278)
Median age, year [IQR]	63.0 [52.0–70.5]	64.0 [56.0–72.0]	63.0 [51.0–70.0]
Male sex, *n* (%)	271 (71.1)	69 (67.0)	202 (72.7)
Positive SARS‐CoV‐2 PCR in LRTs, *n* (%)	128 (33.6)	42 (40.8)	86 (30.9)
Delta variant	39 (35.8)	18 (48.6)	21 (29.2)
Omicron variant	70 (64.2)	19 (51.4)	51 (70.8)
Variant not assessable^a^ (%)	19 (14.8)	5 (11.9)	14 (16.3)
Admission SAPS II^b^ (IQR)	49.5 [37.0–66.0]	50.0 [36.8–67.0]	49.0 [38.0–66.0]
Missing data (%)	19 (5.0)	5 (4.9)	14 (5.0)
Admission NEWS II^c^ (IQR)	9 [7–11]	9 [7–11]	9 [7–11]
Underlying immunosuppression (%)	157 (41.2)	44 (41.7)	118 (41.0)
SOT	52 (13.6)	15 (14.6)	37 (13.3)
HSCT	3 (0.8)	—	3 (1.1)
Hematological malignancy	54 (14.2)	8 (7.8)	46 (16.5)
Cancer	25 (6.6)	8 (7.8)	17 (6.1)
Immunosuppressive treatment	7 (1.8)	1 (1.0)	6 (2.2)
HIV (stage 3)^d^	1 (0.3)	—	1 (0.4)
Neutropenic^e^	52 (13.6)	15 (14.6)	37 (13.3)
HSV‐positive serology (%)	47 (73.4)	17 (100)	30 (63.8)
Missing data	317 (83.2)	86 (83.5)	231 (89.2)

*Note:* Non‐normally distributed continuous variables are expressed as median (IQR). Data are expressed as *n* (%) for qualitative variables.

Abbreviations: COPD, chronic obstructive pulmonary disease; HSCT, hematopoietic stem‐cell transplantation; HSV, Herpes simplex virus; ICU, intensive care unit; NEWS II, national early warning score 2; SAPS II, simplified acute physiology score II; SARS‐CoV‐2, severe acute respiratory syndrome coronavirus 2; SOT, solid organ transplant.

^a^ SARS‐CoV‐2 lineage not assessable due to low viral load and/or degraded RNA preventing determination of full viral sequence.

^b^ Calculated from the worst value of 17 variables collected during the 24 h following admission to intensive care. Possible score from 0 to 163: a high score indicates greater severity [17].

^c^ Calculated from 6 physiological parameters and adjusted to the COPD status of the patients [18]. Score determining the patient acute‐illness severity and prompts critical care intervention. The higher the score, the faster the response needed.

^d^ According to the Centers for the Disease Control and Prevention, 1993 classification.

^e^ Neutrophils <0.5 × 10^9^/L.

—: no occurrence.

#### 3.1.2 Description of the ICU Stay

During the ICU stay, the median duration of mechanical ventilation was 25.0 days (IQR 14.0–45.0) in HSV‐positive patients and 16.0 days (IQR 7.0–29.3) in HSV‐negative (Table 2). A total of 25 patients underwent ECMO; among HSV‐positive patients, the median ECMO duration was 32.0 days (IQR 21.6–58.8), while it was 11.1 days (IQR 3.9–20.0) among HSV‐negative patients.

**Table 2:** Description of the ICU stay.

	Overall population (*n* = 381)	HSV positive (*n* = 103)	HSV negative (*n* = 278)
Oxygenation support			
Duration of mechanical ventilation (days; IQR)	17.0 [8.5–32.0]	25.0 [14.0–45.0]	16.0 [7.0–29.3]
ECMO (%)	25 (6.6)	6 (5.8)	19 (6.8)
Total days on ECMO (IQR)	14.9 [7.0–24.9]	32.0 [21.6–58.8]	11.1 [3.9–20.0]
Immunotherapies (%)			
Corticosteroids	245 (64.3)	73 (70.9)	172 (61.9)
Other^a^	6 (1.6)	1 (0.9)	5 (1.8)
Antiviral treatment (%)			
Acyclovir	88 (23.1)	56 (54.4)	32 (11.5)
Valacyclovir	49 (12.9)	6 (5.8)	43 (15.5)
Preventive	46 (93.9)	4 (66.7)	42 (97.7)
Curative	2 (4.1)	2 (33.3)	—
Not mentioned	1 (2.0)	—	1 (2.3)
Foscarnet	3 (0.8)	2 (1.9)	1 (0.4)
Ganciclovir/valganciclovir	28 (7.3)	7 (6.8)	21 (7.6)
Preventive	26 (92.9)	6 (85.7)	20 (95.2)
Curative	1 (3.6)	1 (14.3)	—
Not mentioned	1 (3.6)	—	1 (4.8)
Anti‐SARS‐CoV‐2 treatments	10 (2.6)	3 (2.9)	7 (2.5)
Convalescent plasma	8 (2.1)	3 (2.9)	5 (1.8)
Paxlovid and remdesivir	2 (0.5)	—	2 (0.7)
Blood stream infection^b^ (%)	162 (42.5)	44 (42.7)	118 (42.4)
ICU length of stay (days; IQR)	24.0 [13.0–44.0]	30.0 [18.0–58.0]	22.0 [11.0–42.3]
ICU mortality (%)	148 (38.8)	41 (39.8)	107 (38.5)

*Note:* Non‐normally distributed continuous variables are expressed as median [IQR]. Data are expressed as *n* (%) for qualitative variables.

Abbreviations: ECMO, extracorporeal membrane oxygenation; HSV, herpes simplex virus; ICU, intensive care unit; Paxlovid, nirmatrelvir and ritonavir; SARS‐CoV‐2, severe acute respiratory syndrome coronavirus 2.

^a^ Corresponding to anakinra, rituximab, ruxolitinib, and tocilizumab.

^b^ Coagulase‐negative *Staphylococcus* bloodstream infection was considered if two sets of blood cultures grew with the same bacteria with the same resistance profile.

—: no occurrence.

Overall, 245 patients (64.3%) received corticosteroids, 73 (70.9%) in the HSV‐positive group and 172 (61.9%) in the HSV‐negative group. Additionally, only 6 (1.6%) patients received tocilizumab, rituximab, anakinra, or ruxolitinib. Regarding anti‐herpetic treatment, 88 patients (23.1%) received ACV, 56 of them (63.6%) were HSV‐positive. Valacyclovir, ganciclovir, and valganciclovir were predominantly used prophylactically in ICU patients. Ten patients (2.6%) received anti‐SARS‐CoV‐2 treatments, including administration of convalescent plasma in 8 of them. The median ICU length of stay of HSV‐positive patients was 30.0 days (18.0–58.0), compared to 22.0 days (IQR: 11.0–42.3) in HSV‐negative patients. The overall ICU mortality rate in the study population was 38.8%.

Among the pathogens co‐isolated in LRTs during the patients' ICU stay, viruses were less prevalent compared to bacteria and fungi. The influenza virus and RSV were isolated in 6 (1.6%) and 7 patients (1.8%), respectively. While no adenovirus was detected, rhinovirus was found in 5 patients (1.3%) and metapneumovirus in 3 (0.8%).

The most frequently isolated bacteria from LRTs were *Pseudomonas aeruginosa* (*n* = 74), *Staphylococcus aureus* (*n* = 55), *Klebsiella spp*. (*n* = 52), *Escherichia coli* (*n* = 32), *Enterobacter cloacae* (*n* = 26), and *Haemophilus influenzae* (*n* = 17). *Candida spp*. and *Aspergillus spp*. were isolated in 211 (55.4%) and 15 patients (3.9%), respectively (Supporting information: Table S1). A total of 162 patients (42.5%) experienced at least one episode of bloodstream infection (Table 2).

### 3.2 HSV Reactivation

Notably, HSV‐1 was found in all HSV‐positive LRTs, while no HSV‐2 was observed. There was a trend toward a higher prevalence of HSV reactivation in the SARS‐CoV‐2 positive group (*p* value = 0.06). Regarding the immune status, among the 128 SARS‐CoV‐2‐positive patients, 65 were immunocompromised (50.8%), and 22 of them (33.8%) were HSV‐positive. Regarding the remaining 63 non‐immunocompromised patients (49.2%), 20 were HSV‐positive (31.7%). In the overall population, of the 253 SARS‐CoV‐2‐negative patients (66.4%), HSV reactivation occurred in 40 (24.8%) out of the 161 non‐immunocompromised patients. Among the 92 immunocompromised and SARS‐CoV‐2‐negative patients, 21 (22.8%) were HSV‐positive. Of note, the prevalence of HSV reactivation was not associated with patients' immune status (OR = 0.97; 95%CI [0.58, 1.60]; *p* value = 0.90; Table 3).

**Table 3:** Univariate and multivariate analyses of factors associated with HSV reactivation.

	Univariate analysis				Multivariate analysis		
	*N*	OR1	95% CI	*p* value	OR1	95% CI	*p* value
Age	381	1.01	1.00; 1.03	0.131	1.01	1.00; 1.03	0.147
Sex	381						
Female		1	—		1	—	
Male		0.76	0.47; 1.25	0.279	0.67	0.40; 1.11	0.118
SARS‐CoV‐2 PCR in LRTs	381						
Negative		1	—		1	—	
Positive		1.54	0.96; 2.45	0.072	1.08	0.56; 2.01	0.824
SARS‐CoV‐2 variant (when PCR is positive)	362						
Omicron		1	—		1	—	
Delta		2.30*	1.01; 5.28	0.047	2.38*	1.01; 5.65	0.048
Underlying immunosuppression	381	1.03	0.65; 1.63	0.896	0.97	0.58; 1.60	0.895
Use of corticosteroids during IC stay	381	1.50	0.93; 2.47	0.104	1.09	0.63; 1.92	0.752
ECMO during IC stay	381	0.84	0.30; 2.06	0.724	0.86	0.26; 2.39	0.785

Abbreviations: CI, confidence interval; ECMO, extracorporeal membrane oxygenation; OR, odds ratio; SARS‐CoV‐2, severe acute respiratory syndrome coronavirus 2.

* *p *< 0.05.

#### 3.2.1 Impact of SARS‐CoV‐2 Variants on HSV Reactivation HSV

Among the 128 SARS‐CoV‐2‐positive patients, viral sequencing was performed in 109 (85.2%). A total of 70 patients (64.2%) were infected by the Omicron variant, of whom 19 patients (27.1%) were HSV‐positive. The remaining 39 patients (35.8%) were infected with the Delta variant, and 18 patients (46.2%) were HSV‐positive. A significantly higher prevalence of HSV reactivation was observed in patients with the Delta variant compared to those with the Omicron variant (*p* value = 0.045). Moreover, among patients infected with the Delta variant, 13 (33.3%) were immunocompromised, whereas 47 (67.1%) were immunocompromised among those infected with the Omicron variant (Table 1 and Supporting Information S1: Figure S1).

The multivariate logistic regression analysis found that only infection with the Delta variant was significantly associated with an increased risk of HSV reactivation; it occurred 2.38 times more frequently in patients infected with the Delta variant compared to those infected with the Omicron variant (95%CI [1.01, 5.65]; *p* value = 0.048; Table 3).

#### 3.2.2 HSV Bronchopneumonia Cytopathological Diagnosis

Among the 381 patients included in the study, only 56 (14.7%) had an available BAL sample for the diagnostic evaluation of herpetic bronchopneumonia. Among them, 14 (25.0%) were HSV‐positive: 5 were SARS‐CoV‐2‐positive, and 9 were SARS‐CoV‐2‐negative. Cytological confirmation of bronchopneumonia was obtained in 3 SARS‐CoV‐2‐positive patients and in 2 SARS‐CoV‐2‐negative patients.

### 3.3 HSV and SARS‐CoV‐2 Viral Loads

Normalization of the HSV viral load was performed in 88 patients (85.4%) among the 103 HSV‐positive patients. A significant correlation was found between normalized and non‐normalized HSV viral loads (*r *= 0.86, 95% CI [0.80, 0.90]; Supporting Information S1: Figure S2). Then, normalization of the SARS‐CoV‐2 viral load was performed in 104 patients (81.3%) among the 128 SARS‐CoV‐2‐positive patients. Hence, among the 42 co‐infected patients, HSV and SARS‐CoV‐2 loads were normalized in 29 (67.4%). Of them, 15 patients (51.7%) had both HSV and SARS‐CoV‐2 normalization in the same sample. In the remaining 14 patients, the time between viral normalizations ranged from 1 to 21 days.

Although it did not reach statistical significance, there was a trend toward lower normalized HSV viral loads in SARS‐CoV‐2‐positive patients compared to negative ones (*p* value = 0.055; Figure 2). In the multivariate linear regression model, two variables were independently associated with normalized HSV viral load corresponding to HSV replication (Table 4). Patients who underwent ECMO during their ICU stay exhibited significantly higher HSV replication compared to those who did not (*β* = 2.8, 95% CI [0.74, 4.80], *p* value = 0.010). Conversely, among SARS‐CoV‐2‐positive patients, higher SARS‐CoV‐2 normalized viral loads were significantly associated with lower HSV replication (*β* = −0.50, 95%CI [–0.87, –0.13]; *p* value = 0.010). Other variables, including age, sex, SARS‐CoV‐2 positivity in the lower respiratory tract, SARS‐CoV‐2 variant type, underlying immunosuppression, and use of corticosteroids—were not significantly associated with HSV replication.

**Figure 2:**
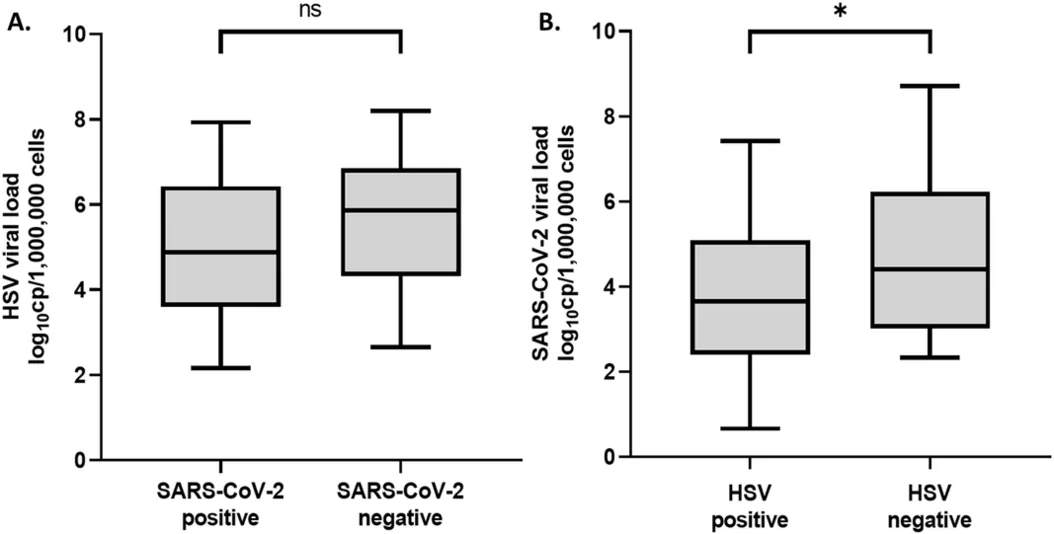
Normalized viral loads in ICU patients. (A) HSV viral load according to the SARS‐CoV‐2 status of patients. (B) SARS‐CoV‐2 viral load according to the HSV status of patients. Viral loads are expressed as base‐10 logarithms and normalized to 10^6^ cells. Data are presented as the median (IQR). *p < 0.05. HSV, herpes simplex virus; SARS‐CoV‐2, severe acute respiratory syndrome coronavirus 2.

**Table 4:** Univariate and multivariate analyses of factors associated with HSV viral load.

	Univariate analysis				Multivariate analysis		
	*N*	Beta	95% CI	*p* value	Beta	95% CI	*p* value
Age	88	0.00	−0.02; 0.02	0.869	0.03	−0.01; 0.06	0.099
Sex	88						
Female		1	—		1	—	
Male		−0.07	−0.53; 0.38	0.751	0.14	−0.49; 0.78	0.644
SARS‐CoV‐2 PCR in LRTs	88						
Negative		1	—		1	—	
Positive		−0.41	−0.83; 0.01	0.054	−0.44	−1.5; 0.66	0.414
SARS‐CoV‐2 variant (when PCR is positive)	33						
Omicron		1	—		1	—	
Delta		0.12	−0.65; 0.88	0.754	−0.28	−1.1; 0.57	0.497
Underlying immunosuppression	88	0.17	−0.26; 0.60	0.434	0.40	−0.35; 1.20	0.283
Use of corticosteroids during IC stay	88	0.12	−0.35; 0.59	0.622	0.38	−0.73; 1.50	0.484
ECMO during IC stay	88	0.87	−0.04; 1.80	0.060	2.8**	0.74; 4.80	0.010
SARS‐CoV‐2 viral load (when PCR is positive)	34	−0.35*	−0.66; −0.03	0.031	−0.50*	−0.87; −0.13	0.010

Abbreviations: CI, confidence interval; ECMO, extracorporeal membrane oxygenation; SARS‐CoV‐2, severe acute respiratory syndrome coronavirus 2.

* *p *< 0.05

** *p *< 0.01.

Regarding SARS‐CoV‐2 replication, the multivariate linear regression model found that HSV reactivation was significantly associated with reduced SARS‐CoV‐2 normalized viral loads (*β *=− 0.89, 95% CI [−1.70, −0.10]; *p* value = 0.028; Table 5 and Figure 2). Other variables, including age, sex, immunosuppression, use of corticosteroids, ECMO during ICU stay, and HSV replication, were not significantly associated with SARS‐CoV‐2 replication.

**Table 5:** Univariate and multivariate analyses of factors associated with SARS‐CoV‐2 viral load.

	Univariate analysis				Multivariate analysis		
	*N*	Beta	95% CI	*p* value	Beta	95% CI	*p* value
Age	98	0.00	−0.03; 0.03	0.965	0.01	−0.02; 0.04	0.484
Sex	98						
Female		1	—		1	—	
Male		0.30	−0.44; 1.00	0.424	0.21	−0.57; 0.98	0.598
HSV PCR in LRTs	98						
Negative		1	—		1	—	
Positive		−0.78*	−1.50; −0.03	0.042	−0.89*	−1.70; −0.10	0.028
SARS‐CoV‐2 variant (when PCR is positive)	84						
Omicron		1	—		1	—	
Delta		−1.1**	−1.90; −0.33	0.006	−0.65	−1.50; 0.19	0.128
Underlying immunosuppression	98	0.85*	0.17; 1.50	0.015	0.66	−0.13; 1.40	0.101
Use of corticosteroids during IC stay	98	−0.51	−1.40; 0.41	0.275	−0.53	−1.70; 0.62	0.361
ECMO during IC stay	98	0.96	−0.39; 2.30	0.161	0.90	−0.57; 2.40	0.228
HSV viral load (centered)	98	−0.30	−0.73; 0.13	0.170	−0.39	−0.82; 0.05	0.078

Abbreviations: CI, confidence interval; ECMO, extracorporeal membrane oxygenation; HSV, herpes simplex virus; SARS‐CoV‐2, severe acute respiratory syndrome coronavirus 2.

* *p *< 0.05

** *p *< 0.01.

### 3.4 Herpetic Antiviral Resistance Testing

#### 3.4.1 Genotypic Analysis

A total of 22 patients (21.4%) were eligible for NGS, since they had two LRTs collected 7 days apart with at least an HSV viral load greater than 5 log copies/mL. Among them, 15 (68.2%) had interpretable sequencing data for both *UL23* and *UL30* on the first LRTs. Regarding *UL23*, one patient harbored T27M classified as USM, while the remaining patients exhibited only polymorphisms. Regarding *UL30*, 3 patients harbored T54K, Q217R, and D741A, respectively, classified as USM.

On the second LRTs, 19 (86.4%) and 17 (77.3%) patients had interpretable sequencing data for *UL23* and *UL30*, respectively. A total of 3 (13.6%) patients harbored a resistance mutation exclusively located on *UL23*. These patients harbored R51W or R220H substitutions, as well as an insertion of a cytosine at position 183. Moreover, on *UL23*, 2 patients (10.5%) also carried P84Q or Q104H classified as USM. On *UL30*, 5 patients (31.3%) harbored T54K, Q217R, M228V, D741A, E756K, respectively, classified as USM.

#### 3.4.2 Phenotypic Analysis

A total of three non‐previously described substitutions were identified in *UL23* and five in *UL30* (Table 6). The three *UL23* substitutions—P84Q, Q104H, and T27M—could not be classified with certainty, as an antivirogram was not feasible. Regarding *UL30*, the phenotypic analysis allowed the classification of the three substitutions Q217R, D741A, and E756K as natural polymorphisms, whereas T54K and M228V substitutions remained of unknown significance due to negative viral cultures.

**Table 6:** Novel *UL23* and *UL30* amino acid substitutions for HSV‐1.

Genes of interest	Substitutions	ACV IC50 (µM)	FOS IC50 (µM)	Classification	Suspected impact
*UL23*					
Non‐conserved regions					
T27M		NP	NP	Unknown	None
Q104H		NA	NA	Unknown	Alteration α‐helix 3 and enzyme structure—potential resistance
Site 2 (AA 83‐88)					
P84Q		NA	NA	Unknown	Alteration of site 2—potential resistance
*UL30*					
Non‐conserved regions					
T54K		NA	NA	Unknown	Potential natural polymorphism
Q217R		1.5	83.0	Natural polymorphism	None
M228V		NA	NA	Unknown	Potential natural polymorphism
D741A		3.5	146.3	Natural polymorphism	None
E756K		0.8	111.9	Natural polymorphism	None

*Note:* The inhibitory concentration 50 (IC50) cut‐off values were 6.5 µM for ACV and 350.0 µM for FOS [11, 19]. The mean IC50 values from two independent experiments are shown for all phenotypically tested substitutions.

Abbreviations: AA, amino acids; ACV, acyclovir; FOS, foscarnet; NA, not assessable (negative viral culture); NP, not performed (insufficient specimen volume for viral culture).

## 4 Discussion

The present retrospective study highlighted a trend toward a greater prevalence of HSV reactivation in LRTs from ICU patients with SARS‐CoV‐2 infection during the 2022 Omicron‐dominant period. Herein, HSV reactivation occurred in 32.8% of SARS‐CoV‐2‐positive patients, which is consistent with a previous report documenting 31% of HSV reactivation in this population [20]. Moreover, the 24.1% prevalence observed among SARS‐CoV‐2‐negative patients in the present study aligns with previous data reporting a prevalence of reactivation ranging from 16% to 27%, depending on the detection method used (viral culture vs. PCR) [21, 22, 23]. To our knowledge, the present study was the first to compare the prevalence of HSV reactivation between patients infected with the Delta variant and those infected with the Omicron variant. A significantly higher prevalence of HSV reactivation was found in patients infected with Delta (46.2%) compared to those with Omicron variant (27.1%). This is consistent with previous studies that reported an HSV reactivation prevalence ranging from 30% to 50% in patients infected with other SARS‐CoV‐2 strains, at the early stages of the pandemic [24, 25]. Of note, the original SARS‐CoV‐2 strain and the Alpha variant, up to 2021, have been associated with a more severe disease and respiratory failure requiring ECMO [26]. Similarly, the Delta variant is known to increase the risk of hospitalization, ICU admission, mechanical ventilation, and mortality compared to Omicron [27], as found herein. The Delta variant infected fewer immunocompromised patients compared to Omicron (33.3% vs. 67.1%) but was associated with a 2.38‐fold increased risk of HSV reactivation. This may be explained by the greater pulmonary tropism of Delta, which likely led to more severe lower respiratory tract infections, as opposed to Omicron, which has a more limited impact on lung tissues [28]. Therefore, since the Delta variant is not currently circulating, the present findings highlight a potential favorable trend, given the fewer pulmonary HSV reactivations in ICU patients infected with Omicron.

While no clear evidence of direct viral interference was observed herein, the 1 log reduction in SARS‐CoV‐2 replication among HSV‐positive patients may reflect the natural course of SARS‐CoV‐2 infection, with a progressive decline in SARS‐CoV‐2 replication over time, followed by the HSV reactivation. Importantly, this temporal sequence suggests that HSV reactivation occurs at a stage when the primary SARS‐CoV‐2 infection is expected to decline and may therefore represent a marker of SARS‐CoV‐2–induced immune dysregulation. This immune dysregulation is characterized by a high cytokine response (including IL‐6, IL‐8, IL‐10, and TNF‐α) along with impaired antiviral immunity driven by reduced type I and III interferon responses. It is also associated with T and NK lymphopenia, exhaustion of CD4+ and CD8+ T cells, and IL‐6–mediated neutrophil‐driven suppression of T lymphocytes via increased PD‐1/PD‐L1 signaling. Furthermore, alterations of the innate immune system including neutrophilia, decreased HLA‐DR expression on CD14+ monocytes, and reduced MHC class II expression, allows a permissive environment for secondary infections [29]. It is also potentially linked to ICU‐related immunoparalysis and to epithelial injury due to mechanical ventilation [2, 5]. In the present study, ECMO was associated with significantly higher HSV viral loads in the LRTs, 3 log copies/10^6^ cells greater, compared to non‐ECMO patients. However, this association is highly limited, given the very small number of patients who underwent ECMO (*n* = 6), and the potential temporal bias, since HSV reactivation occurred after ECMO discontinuation in two cases. Beyond these limitations, it remains unclear whether ECMO itself contributes to HSV reactivation or whether this association simply reflects the severity of underlying disease. Larger prospective studies are needed to confirm these findings and to determine whether routine monitoring of HSV reactivation in ECMO patients might be warranted.

One of the key findings herein was the high prevalence of HSV resistance to antiherpetic treatment, reaching at least 13.6% in the present cohort. This resistance prevalence is consistent with that reported in the general immunocompromised population [30, 31]. Furthermore, due to the presence of two substitutions, P84Q and Q104H, which are strongly suspected of inducing ACV resistance, this prevalence could be even higher, potentially reaching 22.7%. In fact, the location of these substitutions has been described as conferring ACV resistance, although the amino acids identified herein differ from those in previous reports [32, 33, 34]. As expected, these mutations were exclusively located in *UL23*, encoding the viral thymidine kinase [35]. This finding indirectly supports the notion that ICU patients may be considered as secondarily immunocompromised, particularly in the context of immunoparalysis [5]. This also underscores the need for a systematic screening of HSV antiviral susceptibility in this high‐risk population, in whom resistance is prone to emerge. Resistance screening appears particularly relevant around Day 7 following treatment initiation, as resistant HSV strains in immunocompromised patients often emerge between Days 7 and 14 of treatment [35]. The NGS method used in the present study identified eight substitutions that had not been previously reported in the literature. These USM can hinder the diagnosis of HSV resistance to antivirals. Phenotypic antiviral susceptibility testing allowed us to classify three of them, Q217R, D741A, and E756K, as natural polymorphisms. In vitro characterization of these substitutions should be performed using methodologies including the generation of recombinant HSV strains through cosmid or plasmid assembly, or CRISPR/Cas9‐mediated site‐directed mutagenesis [36, 37].

However, the present study has several limitations. First, there is a lack of data regarding the timing of COVID‐19 symptom onset, and a longitudinal follow‐up was not performed, which would have been necessary to assess potential viral interferences. While interactions between respiratory viruses have been described, it would be valuable to investigate interactions between respiratory viruses and other viruses, such as herpes viruses, which may reactivate due to immunosuppression [38, 39, 40]. The present study also highlighted the lack of data regarding herpetic bronchopneumonia, underscoring a key challenge in differentiating HSV reactivation from true bronchopneumonia. Since the study was not designed to compare cytopathological and PCR data, these findings should be interpreted with caution. Herein, the cytopathological evaluation, which is a useful tool for diagnosis, was performed in only 15% of HSV‐positive patients since it is not included in the management protocol. This likely led to an underestimation of the true prevalence of bronchopneumonia, thereby exposing patients to a poorer prognosis with an increased risk of mortality. Conversely, the prevalence of cytologically confirmed bronchopneumonia is expected to be lower than that of HSV reactivation, as reactivation may occur in the absence of herpetic nuclear inclusions. This gap underscores the need to adapt current practices, either by promoting more systematic evaluation of LRTs for pathology or by developing new diagnostic criteria that do not exclusively rely on cytopathological confirmation.

These findings highlight the potential role of SARS‐CoV‐2 variant, particularly Delta, in increasing HSV reactivation risk in the ICU. The pattern observed is consistent with primary SARS‐CoV‐2 infection followed by secondary HSV reactivation, making the interpretation of viral loads challenging. ECMO‐related findings are exploratory and should be interpreted cautiously, requiring validation in larger cohorts. The high prevalence of antiviral resistance further emphasizes the need for systematic evaluation of HSV susceptibility in ICU patients, who are particularly prone to the emergence of resistance.

## Author Contributions

Bastien Zarlenga, Emilie Frobert, Jean‐Christophe Richard, and Florent Wallet contributed to the study concept and design, as well as to data analysis and interpretation. Gregory Destras contributed to the phylogenetic analysis of SARS‐CoV‐2 genomes. Christophe Ginevra, Bruno Simon, and Jennifer Giorgi provided bioinformatics support for next‐generation sequencing data analysis. Muriel Rabilloud and Salomé Avenas performed and interpreted the statistical analyses. Alexis Trecourt provided cytopathological data from BALs. Florence Morfin, Alexandre Gaymard, Geneviève Billaud, and Anne‐Claire Lukaszewicz contributed to manuscript review and editing.

## Funding

The authors have nothing to report.

## Ethics Statement

Data storage and data analysis complied with legal and ethical standards, thereby safeguarding the privacy and rights of individuals whose data was being used (MR004 methodology as appropriate for French study; registration number 23‐5167). This study has been approved by the institutional review board of the *Hospices Civils de Lyon* on July 6, 2023.

## Consent

In accordance with the current French law, non‐opposition was obtained from all included patients.

## Conflicts of Interest

Florent Wallet reports receiving congress fees from Philips Healthcare and Jazz Pharma, and lecture fees from Kite (Gilead) and Novartis. Anne‐Claire Lukaszewicz is a member of the joint research laboratory between bioMérieux and Hospices Civils de Lyon, and has participated in symposia organized by AOP, Viatris, and Chiesi. Jean‐Christophe Richard reports receiving congress fees from Pfizer and lecture fees from AstraZeneca. The other authors declare no conflicts of interest.

## Supporting information


Supporting File

## Data Availability

All Illumina sequences have been deposited in the European Nucleotide Archive database (accession number PRJEB79652).
