# Supplementary material for: Pulmonary Herpes Simplex Virus Reactivation in Invasively Mechanically Ventilated Patients With COVID‐19 in Intensive Care Unit
Source: J Med Virol. 2026 Oct 7;98(10):e71170. doi: 10.1002/jmv.71170 (PMC13644695; doi:10.1002/jmv.71170)
Supplement: Supplementary file 1 [file JMV-98-e71170-s001.docx]

Supporting Information.

Table S1. Co-isolations in LRTs.

| **Pathogens** | | | | **Overall population**  **(n= 381)** | **HSV positive**  **(n=103)** | **HSV negative**  **(n=278)** |  |  |
| --- | --- | --- | --- | --- | --- | --- | --- | --- |
| **Viruses (%)** | | | |  |  |  |  |  |
|  | *RSV* | | | 7 (1.8) | - | 7 (2.5) |  |  |
|  | *Influenza virus* | | | 6 (1.6) | 1 (1.0) | 5 (1.8) |  |  |
|  |  | | Missing data | 70 (18.4) | 19 (18.4) | 51 (18.3) |  |  |
|  | *Rhinovirus* | | | 5 (1.3) | 1 (1.0) | 4 (1.4) |  |  |
|  | *Metapneumovirus* | | | 3 (0.8) | - | 3 (1.1) |  |  |
|  | *Adenovirus* | | | - | - | - |  |  |
|  |  | | Missing data | 99 (26.0) | 25 (24.3) | 74 (26.6) |  |  |
| **Bacteria (%)** | | | |  |  |  |  |  |
|  | *Pseudomonas aeruginosa* | | | 74 (19.4) | 25 (24.3) | 49 (17.6) |  |  |
|  | *Staphylococcus aureus* | | | 55 (14.3) | 12 (11.7) | 43 (15.5) |  |  |
|  |  | | MRSA | 6 (10.9) | 2 (16.7) | 4 (9.3) |  |  |
|  | *Klebsiella spp.* | | | 52 (13.6) | 15 (14.6) | 37 (13.3) |  |  |
|  | *Escherichia coli* | | | 32 (8.4) | 12 (11.7) | 20 (7.2) |  |  |
|  | *Enterobacter cloacae* | | | 26 (6.8) | 9 (8.7) | 17 (6.1) |  |  |
|  | *Haemophilus influenzae* | | | 17 (4.5) | 6 (5.8) | 11 (4.0) |  |  |
|  | *Stenotrophomonas maltophilia* | | | 13 (3.4) | 4 (3.9) | 9 (3.2) |  |  |
|  | *Citrobacter spp.* | | | 13 (3.4) | 5 (4.9) | 8 (2.9) |  |  |
|  | *Streptococcus pneumoniae* | | | 12 (3.1) | 5 (4.9) | 7 (2.5) |  |  |
|  | *Proteus spp.* | | | 9 (2.4) | 3 (2.9) | 6 (2.2) |  |  |
|  | *Enterococcus spp.* | | | 9 (2.4) | 3 (2.9) | 6 (2.2) |  |  |
|  | *Serratia marcescens* | | | 5 (1.3) | 2 (1.9) | 3 (1.1) |  |  |
|  | *Legionella pneumophila* | | | 4 (1.0) | 1 (1.0) | 3 (1.1) |  |  |
|  | *Hafnia alvei* | | | 4 (1.0) | 2 (1.9) | 2 (0.7) |  |  |
|  | *Acinetobacter spp.* | | | 4 (1.0) | 2 (1.9) | 2 (0.7) |  |  |
|  | *Morganella morganii* | | | 3 (0.8) | 3 (2.9) | - |  |  |
|  | *Staphylococcus epidermidis* | | | 2 (0.5) | 2 (1.9) | - |  |  |
|  | Others | | | 17 (4.5) | 4 (3.9) | 13 (4.7) |  |  |
|  |  | Missing data | | - | - | - |  |  |
| **Fungi (%)** | | | |  |  |  |  |  |
|  | *Candida spp.* | | | 211 (55.4) | 61 (59.2) | 150 (54.0) |  |  |
|  | *Aspergillus spp.* | | | 15 (3.9) | 6 (5.8) | 9 (3.2) |  |  |
|  | *Pneumocystis jirovecii* | | | 2 (0.5) | 1 (1.0) | 1 (0.4) |  |  |
|  |  | Missing data | | - | - | - |  |  |

Data are expressed as n (%).

Others: *Achromobacter spp., Bordetella spp, A.odotolyticus, C.striatum, M.catarrhalis, N.mucosa, S.agalactiae, S.anginosus, S.constellatus, S.pyogenes, Sphingomonas spp., Raoultella spp.*.

HSV: Herpes simplex virus; LRTs : lower respiratory tract samples; MRSA: methicillin-resistant *Staphylococcus aureus*; RSV: Respiratory syncytial virus.

**Figure S1. Phylogenetic analysis of SARS-CoV-2 genome from ICU patients.**

Phylogenetic tree was constructed with all SARS-CoV-2 available sequences compared to the original SARS-CoV-2 sequence as a reference (highlighted in bold black; GenBank accession number MN908947.3) using IQ-TREE online tool (model selection and tree inference). Variant were labeled according to their Nextstrain nomenclature. Each color indicates one clade: navy, turquoise, violet, green, light green, olive green, and orange for 21A, 21J, 21I, 21M, 21L, 22B, and 21K clades, respectively. Circles correspond to patients who experienced respiratory herpes reactivation. Patient samples are identified by anonymized numbers. Scale (bottom) represents the number nucleotide substitutions in the whole genome.

**Figure S2. HSV Viral load measurements expressed as copies per mL and copies per 1,000,000 Cells.**

A. Violin plots representing log_10_ HSV viral loads. Median and interquartile range are represented by the thick and the thinner lines, respectively. B. Correlation between HSV normalized and non-normalized viral loads. The Spearman correlation test revealed an R value of 0.86 (95%CI = 0.80 – 0.90).

HSV: Herpes simplex virus.
